# Ubiquitin pathways in neurodegenerative disease

**DOI:** 10.3389/fnmol.2014.00063

**Published:** 2014-07-08

**Authors:** Graham Atkin, Henry Paulson

**Affiliations:** Department of Neurology, University of MichiganAnn Arbor, MI, USA

**Keywords:** ubiquitin, neurodegenerative diseases, proteasome, protein quality control, Alzheimer's disease, Parkinson disease, Amyotrophic Lateral Sclerosis, Huntington's disease

## Abstract

Control of proper protein synthesis, function, and turnover is essential for the health of all cells. In neurons these demands take on the additional importance of supporting and regulating the highly dynamic connections between neurons that are necessary for cognitive function, learning, and memory. Regulating multiple unique synaptic protein environments within a single neuron while maintaining cell health requires the highly regulated processes of ubiquitination and degradation of ubiquitinated proteins through the proteasome. In this review, we examine the effects of dysregulated ubiquitination and protein clearance on the handling of disease-associated proteins and neuronal health in the most common neurodegenerative diseases.

## Introduction

The unique demands placed on neurons by their exquisitely complicated and dynamic architecture have been appreciated by investigators for over 100 years (Golgi, 1886; Ramon y Cajal, 1909; Herculano-Houzel, 2011). A recent study places the number of neurons in the human brain at approximately 85 billion (Azevedo et al., 2009). With each neuron making upwards of 10 thousand synaptic connections, the estimated total number of synapses reaches toward 8.5 hundred trillion (8.5 × 10^14^). Astonishingly, plasticity can occur selectively at particular subsets of synapses within a neuron, even down to the level of a single specified synapse (Lee et al., 2010). Failure to maintain these synaptic connections and their proper plasticity are hallmarks of a host of neurodegenerative diseases, and loss of synaptic connections correlates with diminished cognitive function even before neurons degenerate (Masliah et al., 1991a,b). With 2788 unique proteins already identified as integral to the composition of each synapse (Pielot et al., 2012), the management of unique protein environments requires sufficiently complex and modifiable systems of protein quality control. The Ubiquitin Proteasome System (UPS), a set of interacting enzymes and associated proteins, is able to address these diverse proteostatic needs through the orchestrated activity of over 500 components working in versatile combinations to regulate protein-protein interactions and eliminate unwanted proteins. As newly synthesized proteins form new structures and connections, the UPS works to ensure that old proteins are degraded to make way and that the proper complement of building materials is available. To achieve these functions, components of the UPS are recruited to dendritic spines in response to synaptic activity (Bingol and Schuman, 2006; Bingol et al., 2010). Evidence continues to mount for the necessity of UPS involvement in the dynamic remodeling of synaptic structures following synaptic activity (Hegde et al., 1997; Ehlers, 2003; Pak and Sheng, 2003; Patrick et al., 2003; Bingol and Schuman, 2004; Hung et al., 2010; Fu et al., 2011). Many synaptic components have been identified as activity-dependent substrates for agents of the UPS. These include postsynaptic receptors (Kato et al., 2005; Lussier et al., 2011) and the scaffolding proteins which hold them in place (Colledge et al., 2003; Shin et al., 2012). Transsynaptic proteins such as Beta-Catenin and Beta-Integrin align and connect pre- and post-synaptic elements and are degraded through the UPS (Yoshida et al., 2002; Dreier et al., 2005). The shape, size, and placement of dendritic spines are also determined in part by proteins that regulate cytoskeletal organization and are themselves subject to ubiquitin-mediated degradation (Bingol and Schuman, 2005; Marland et al., 2011). These contributions to synapse maintenance and synaptic plasticity require that the UPS functions properly. For example, pharmacologic inhibition of the UPS leads to a frank reduction in activity-dependent synaptic plasticity (Ehlers, 2003) and a dose-dependent loss of synaptic connections (Bajic et al., 2012). Robust loss of synaptic connections is evident in all of the major neurodegenerative disorders. The full role of ubiquitination pertaining to synaptic structure and function remains incompletely understood, but has been the focus of significant investigation (Hegde and Upadhya, 2007; Bingol and Sheng, 2011; Hanus and Schuman, 2013).

As important as the contributions of the UPS to maintaining the plasticity of synapses are its diverse roles in ensuring general cell health, including the elimination of misfolded or damaged proteins, mediation of receptor signaling pathways, response to DNA damage and oxidative stress, and progression of the cell cycle, among other roles (Bernassola et al., 2010; Shang and Taylor, 2011). UPS function is essential to cell health and survival in all cell types. Improper clearance of proteins is believed to be a causative or contributing factor in many neurodegenerative diseases, which are often characterized by the accumulation of aggregated proteins (Alves-Rodrigues et al., 1998; Huang and Figueiredo-Pereira, 2010). Whether aggregation itself is the cause of toxicity or merely represents a strategy by which neurons sequester toxic proteins remains contested (Ross and Poirier, 2005), but the failure of quality control pathways to eliminate these unwanted proteins is evident. UPS dysfunction has been reported in the most common neurodegenerative diseases, including Alzheimer's disease (AD), Parkinson's disease (PD), Amyotrophic Lateral Sclerosis (ALS), and Huntington's disease (HD), as well as less common disorders and various animal models of protein aggregation (Keller et al., 2000; Bence et al., 2001; McNaught et al., 2003; Seo et al., 2004; Lonskaya et al., 2013). How the UPS becomes impaired in disease states is not always clear, as too little is known about how disease-related proteins are handled under normal conditions. Research into these areas has begun to reveal just how intricate and extensive ubiquitin-mediated processes within the cell are, and accordingly, just how significant their dysregulation in a pathogenic context must be. Recent studies describing the plurality of ubiquitin-related abnormalities seen in disease states suggest the intriguing possibility that the disruption of one such process leads into that of another. Hopefully, further investigations will uncover potential therapeutic interventions.

## Ubiquitination and the ubiquitin proteasome system

**Figure 1:**
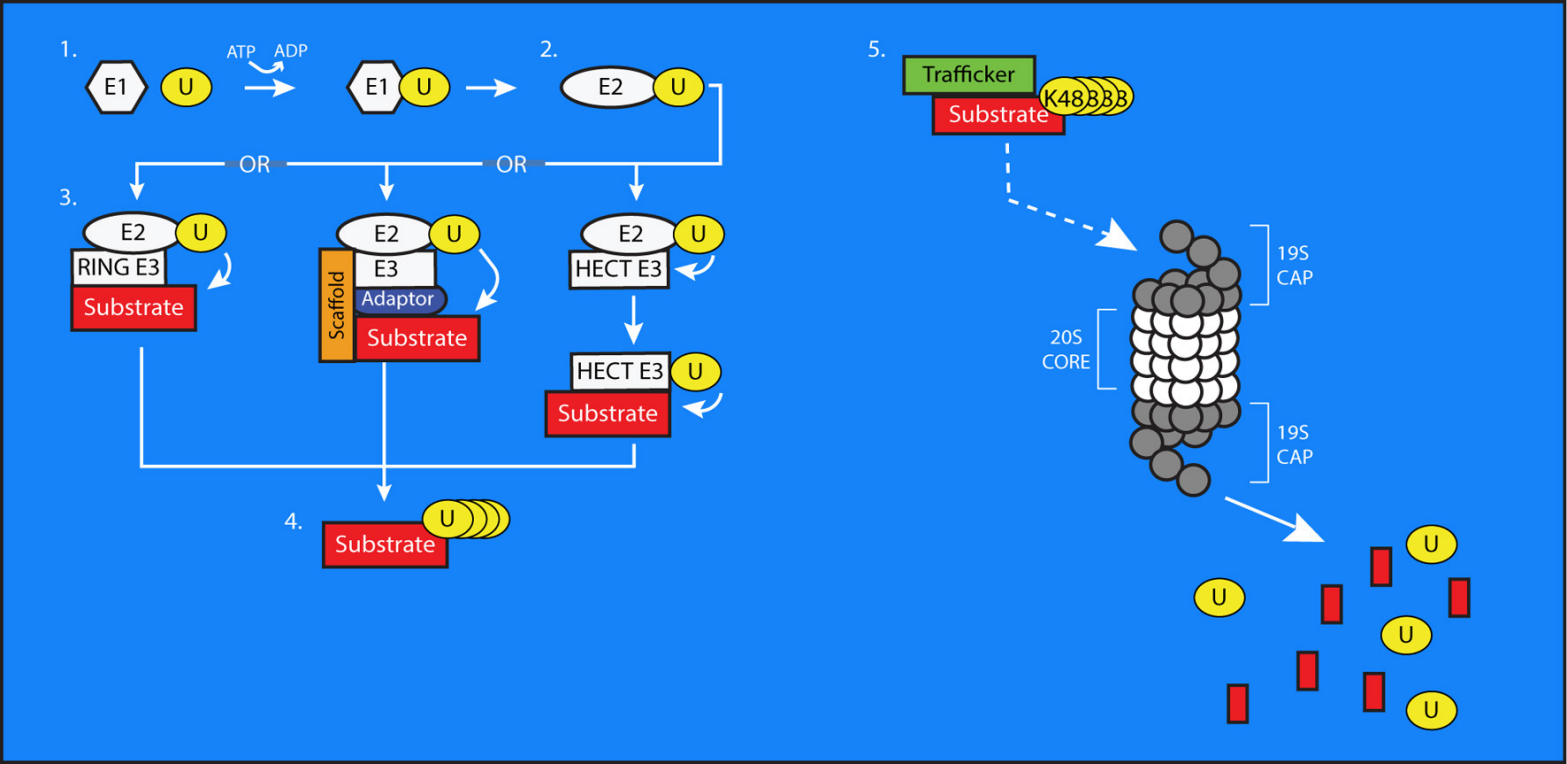
**The process of ubiquitin conjugation**. 1. Ubiquitin (U) is bound via a thioester bond to the active-site cysteine of an E1 Ubiquitin-Activating enzyme, through a process requiring ATP. 2. The ubiquitin molecule is then passed to an E2 Ubiquitin-Conjugating Enzyme through trans(thio)esterification. 3. An E3 ubiquitin ligase brings the E2 into sufficiently close proximity and correct alignment with a substrate protein to facilitate the transfer of ubiquitin to a target residue. In the case of HECT-type E3s, the ubiquitin is first transferred to an active site cysteine on the E3 before being conjugated to the substrate. E3s can also exist as multi-subunit complexes including scaffolding and adaptor proteins that confer substrate specificity to the process of ubiquitin transfer. 4. Additional ubiquitin molecules can be added onto the first to create polyubiquitin chains on substrate proteins. 5. The 26S proteasome is composed of a cylindrical, proteolytic 20S core which is capped at both ends by a 19S regulatory cap. Polyubiquitinated substrate proteins, typically bearing K48-linked chains, are targeted to the proteasome by trafficking proteins. The proteasome digests these substrates into smaller peptides and free ubiquitin molecules, which then can be used to modify further substrates.

Ubiquitin is a 76 amino acid (~8 kDa) protein expressed in all eukaryotic cells. It is highly conserved throughout evolution; the amino acid sequence of human ubiquitin is identical to that of Aplysia (Hegde et al., 2000) and nearly identical to that of yeast (Finley and Chau, 1991). Through the coordinated activity of multiple enzymes, ubiquitin is covalently added to substrate proteins through the formation of an iso-peptide bond between the C-terminal diglycine motif of ubiquitin and lysine residues on the target (Figure 1) (Hershko and Ciechanover, 1998). This cascade begins with the ATP-dependent attachment of ubiquitin's C-terminal glycine through a thio-ester bond to an active-site cysteine on an Ubiquitin Activating Enzyme (E1). This ubiquitin is then transferred to a Ubiquitin Conjugating Enzyme (E2) through a thio-ester bond. From there, the E2 enzyme will cooperate with a Ubiquitin Ligase (E3) to transfer the ubiquitin molecule to the target lysine on a substrate protein. This last step occurs differently depending on the type of E3 involved, but it is at this step that substrate specificity is believed to occur, with E3 ligases selecting substrates for ubiquitination. However, this view has recently come under some scrutiny, as evidence emerges for a role for E2s in the process of substrate selection (Scaglione et al., 2013).

E3s are typically grouped into three classes, with each defined by the specific protein domains it possesses. These domain-based classes include: (1) Really Interesting New Gene (RING) finger-containing E3s, (2) Homologous to E6-AP (HECT) domain-containing E3s, and (3) E3s composed of multiple subunits. RING finger-containing E3s bring the E2 and the substrate protein into sufficiently close proximity for the transfer of the ubiquitin to its target lysine (Lorick et al., 1999). HECT-domain containing proteins possess an active-site cysteine to which the ubiquitin is first transferred from the E2 before being passed to the substrate protein (Huibregtse et al., 1995). In contrast to these single-unit E3 ligases, multi-subunit ligases are composed of multiple adaptor proteins, cofactors, and scaffolding proteins that confer substrate specificity and facilitate ubiquitination (Cardozo and Pagano, 2004). The Skp1/Cul1/F-box (SCF) protein complex and the Anaphase-promoting Complex (APC) are among the best studied of these multi-subunit E3s. SCF complexes can include various combinations of scaffolding proteins called cullins, F-box proteins, and substrate adaptors (Hao et al., 2005). APC is less variable, containing Apc2, Apc11, and either Cdh1 or Cdc20 for substrate recognition (Biggs et al., 2006). Each E3 can recognize multiple substrates; Fbxw1/β-TRCP1, for example, has upwards of 40 documented substrates itself (Skaar et al., 2009). Further contributing to the complexity of these multi-subunit E3 ligases is the developmental and spatial restriction of their expression within cells (Pines, 2006). There are estimated to be at least 500 different E3 ligases, with more continuing to be discovered (Ardley and Robinson, 2005).

This highly regulated process adds ubiquitin to target lysines on the substrate, but it can also add other ubiquitin molecules onto lysines of a ubiquitin already conjugated to a substrate. By this process, ubiquitin chains of varying length and composition can be formed. The elongation of ubiquitin chains can occur at any of ubiquitin's own seven lysines, resulting in the formation of different linkage types (Peng et al., 2003). Although all possible linkage types are present in cells, their precise functions remain only partially understood (Xu et al., 2009). Chains formed through the addition of ubiquitin exclusively at lysine 48 (K48) have been recognized to signal protein degradation (Glickman and Ciechanover, 2002), whereas K63-linked ubiquitin chains seem to subserve diverse functions beyond protein degradation (Jacobson et al., 2009). For example, K63-linked chains regulate NF-κB signaling not by promoting protein degradation but by influencing ubiquitin-dependent protein-protein interactions (Hadian et al., 2011). Elsewhere, however, they have been implicated in promoting the lysosomal degradation of the low density lipoprotein receptor (LDLR) (Zhang et al., 2013) and the epidermal growth factor receptor (EGFR) (Huang et al., 2013). Both K48- and K63-linked chains have been observed to modify kinase activity in response to cellular stress, as have K11-linked chains (Ben-Neriah, 2002; Bertrand et al., 2011). K11-linked chains are critical for cell-cycle regulation and cell division (Matsumoto et al., 2010). Other linkages, including atypical, mixed-type linkages are less well studied, but have been implicated in similar processes within the cell (Ikeda and Dikic, 2008; Husnjak and Dikic, 2012).

The functions thus far attributed to specific chain linkages represent only a fraction of the diverse roles ubiquitin is known to play in cellular processes (Husnjak and Dikic, 2012), many of which do not depend on proteasome function (Mukhopadhyay and Riezman, 2007). Ubiquitination regulates DNA repair (Jackson and Durocher, 2013), protein localization and endocytosis (Haglund et al., 2003; Hicke and Dunn, 2003; Schnell and Hicke, 2003), and protein-protein interactions (Hoege et al., 2002; Moldovan et al., 2007). Free, unanchored chains of ubiquitin molecules are also present in cells and can regulate numerous functions including kinase activation (Xia et al., 2009). The UPS has been also been implicated in the turnover of mRNA, although whether this regulation is direct or indirect remains unclear (Cano et al., 2010). Intriguingly, E3 ligases can themselves be targeted for ubiquitination, offering an additional level of control for this important pathway (Buschmann et al., 2000; Xiong et al., 2009). The ubiquitination of E3 ligases can result either in their degradation or in the modification of their activity (Scaglione et al., 2011).

Once a substrate is tagged for elimination through the addition of a ubiquitin chain, it must be targeted to the cell's degradation machinery by chaperone proteins that recognize and bind to poly-ubiquitin chains. One such chaperone is Valosin-containing protein (VCP), which has been shown to physically interact with and shuttle poly-ubiquitinated substrates to the proteasome to facilitate their degradation (Figure 1) (Dai and Li, 2001). The proteasome contains a catalytic protein complex referred to as the 20S core (Lowe et al., 1995), which is capped at each end by a regulatory protein complex (19S) (Finley, 2009; Zhang et al., 2009). It is responsible for breaking down substrate proteins into small peptides (Hough et al., 1987; Hadari et al., 1992).

The ubiquitination of substrate proteins is a reversible process. The removal of ubiquitin is carried out by De-ubiquitinating Enzymes (DUBs). DUBs play two important roles, allowing for the editing of existing chains (Wilkinson, 1997) and the removal of ubiquitin chains altogether from a substrate. As the presence of ubiquitin chains prevents substrates from entering the proteasome due to spatial restrictions, DUBs play an essential role in determining the rate of protein clearance in cells (Hershko and Ciechanover, 1998; Wilkinson, 2009), and certain DUBs including USP14 are known to associate directly with the19S regulatory complex of the proteasome (Borodovsky et al., 2001).

It is important to consider that while numerous studies report proteasome dysfunction in disease states, proteasomal degradation is only one of several potential outcomes of ubiquitination that may contribute to pathogenesis when impaired. Indeed, there are numerous examples of dysregulated protein handling due to failures in ubiquitination that do not necessarily implicate the proteasome. Whether the effects of proteasomal failure reach upstream to impact the activity and efficacy of E1s, E2s, or E3s is not clear. Given that K48-linked chains are among the most common found in cells (Ziv et al., 2011), it is conceivable that impaired proteolysis could deplete cellular pools of free ubiquitin and thereby induce further dysfunction. Accordingly, an imbalance in ubiquitination/deubiquitination activities may result in improper chain formation, preventing the proteasome from recognizing and handling targeted substrates. To examine these two separate yet related phenomena, it will be helpful to distinguish whether dysfunction occurs in UPS *per se* or, more broadly in the Ubiquitin Signaling System (USS) which includes the labeling of proteins with ubiquitin for any number of intended outcomes beyond degradation. While impaired clearance characterizes UPS failure, it remains to be seen whether and how the other, non-proteasomal components of the USS contribute to disease processes. One example of this distinction is evident in the ubiquitin-mediated fate of Caspase proteins.

Though the precise methods by which neurons degenerate in disease remain unclear, substantial evidence supports a role for proteases of the Caspase family. For example, activated caspases are elevated in AD patient tissue (Bredesen, 2009). Upon activation, caspases can either activate other proteases (initiator caspases) or damage essential components of the cell and promote apoptosis (effector caspases). Sublethal amounts of caspase activation have been linked to synaptic dysfunction in an animal model of AD (D'Amelio et al., 2011). Caspase-mediated effects can be inhibited both by the inactivation of caspase enzyme activity and by their targeted degradation through the proteasome. The X-linked Inhibitor of Apoptosis Protein (XIAP) and its family member cIAP1 are RING-type E3 ligases that directly bind to and target caspases for degradation; XIAP is also able to inhibit the proteolytic activity of caspases (Suzuki et al., 2001; Choi et al., 2009). The regulation of caspases by XIAP is limited, however, in oxidative stress conditions. Both acute and chronic inflammation, which are often associated with disease, elevate the levels of nitric oxide in neurons. This elevation can lead to the aberrant addition of nitric oxide to proteins (nitrosylation). Dysregulated nitrosylation of proteins is evident in several neurodegenerative diseases, including AD (Nakamura et al., 2013). The addition of nitric oxide (nitrosylation) to the active cysteine of XIAP's RING domain inactivates its E3 ligase activity. Nitrosylated XIAP is unable to ubiquitinate caspases and thus unable to inhibit apoptosis (Nakamura et al., 2010). The relative amount of nitrosylated XIAP is increased in AD patient tissue, suggesting a role in the accelerated apoptosis observed in disease (Nakamura et al., 2010). Here, then, is one example in which the state of the proteasome in disease is secondary to the decreased ability of E3 ligases to target caspases for degradation. Therefore, it is essential to consider how disease-related proteins are modified by agents of the USS while also examining whether affected neurons can execute the intended consequences of that modification.

## Ubiquitination in neuropathology

To further illustrate the complexity and fundamental significance of ubiquitin-mediated pathway involvement, we review key findings about the handling of disease-related proteins by the USS and UPS in several of the most common neurodegenerative diseases.

### Alzheimer's disease

**Figure 2:**
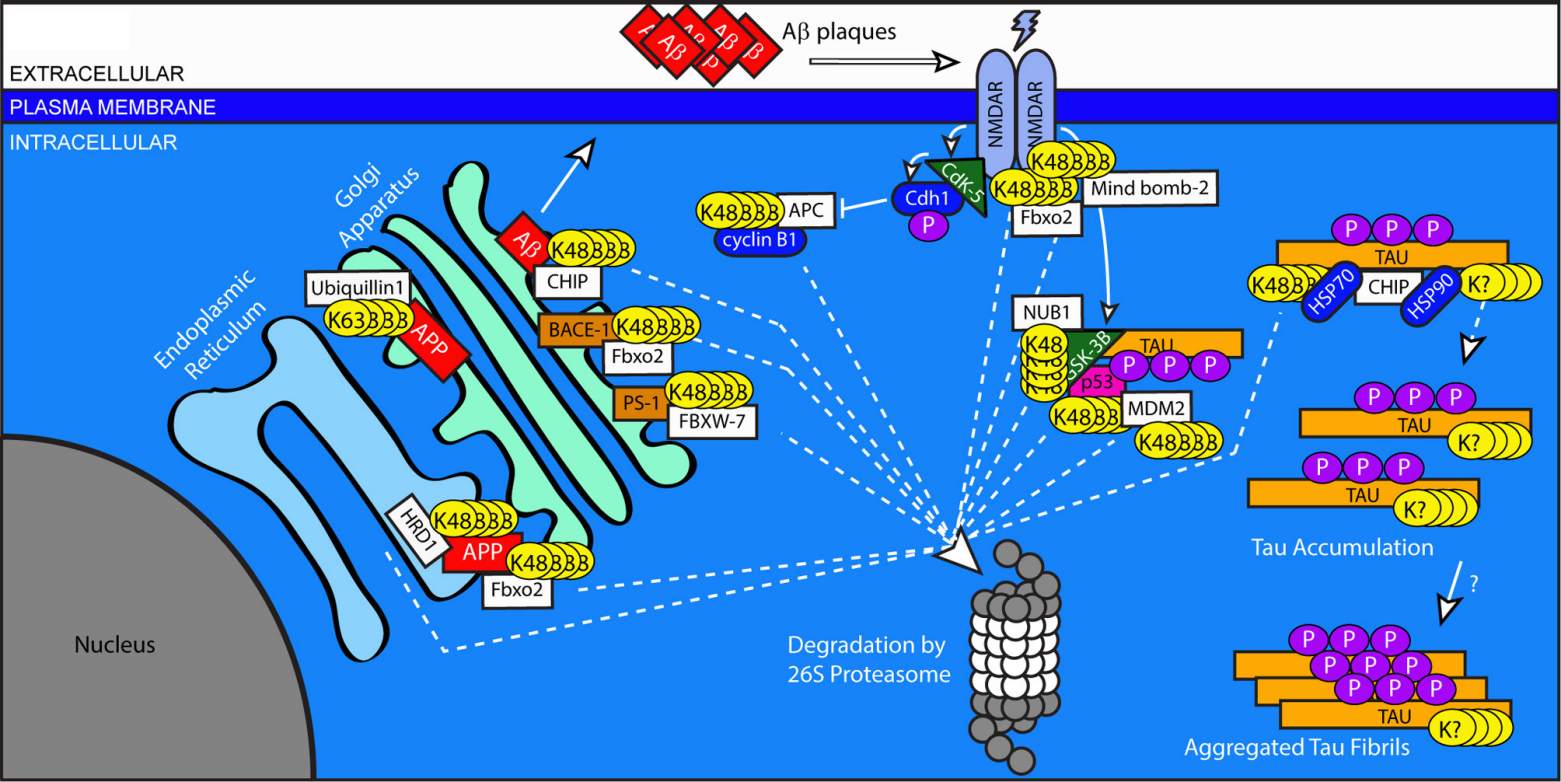
**Regulation of protein trafficking, receptor signaling, and protein clearance by ubiquitination in Alzheimer's disease**. Improper processing of APP and Tau contribute to the pathology of AD. Excessive or improperly folded APP is cleared from the ER by HDR1 and Fbxo2 through the addition of K-48 linked chains. Ubiquitin-mediated processes are indicated by dashed lines. Maturation of APP is arrested in the early secretory pathway by non-degradative, K-63 linked ubiquitination that is stimulated by Ubiquilin 1. Surface APP is endocytosed to the late Golgi, where it is cleaved by secretases including BACE-1 and PS-1, whose levels are regulated by the E3 ligases Fbxo2 and FBXW-7, respectively. The cleavage of APP results in the production of Amyloid-Beta, which can be targeted for degradation by CHIP. Uncleared Amyloid-Beta is exocytosed to the extracellular space, where it aggregates to form plaques. Amyloid-Beta can influence NMDA receptor signaling. NMDA receptor activation stimulates the kinase Cdk5, which results in the downstream inhibition of the E3 ligase APC and blocks the degradation of cyclin B1. NMDA receptor signaling also increases the activation of GSK3B, which phosphorylates Tau. GSK3B is targeted for degradation by NUB1, which also blocks the interaction between GSK3B and Tau. GSK3B activity is increased by complexing with p53, whose levels are regulated by the E3 ligase MDM2. Under conditions of cellular stress, MDM2 auto-ubiquitinates and targets itself for degradation. The E3 ligase CHIP targets Tau, but can have divergent effects on its handling. When working in combination with Hsp70, CHIP targets Tau for degradation. However, when Hsp90 is involved, CHIP facilitates an alternative ubiquitination of unknown linkage type, resulting in the accumulation of phosphorylated tau. Through an unknown mechanism, this accumulated Tau then forms insoluble protein aggregates.

Alzheimer's disease is the most common form of dementia and the most common neurodegenerative disorder. Its symptoms include a progressive decline in memory and other cognitive functions. Histologically, AD involves extensive neurodegeneration and loss of synaptic connections, resulting in progressive atrophy of the temporal, frontal and parietal lobes of the cerebral cortex. It is characterized by the hallmark deposition of intracellular, filamentous aggregates mainly consisting of hyper-phosphorylated Tau (neurofibrillary tangles, NFTs) and extracellular plaques rich in Amyloid-Beta (amyloid plaques) (Glenner et al., 1984; Goedert et al., 1988; Wischik et al., 1988). Ubiquitinated forms of Tau and Amyloid-Beta, as well as other ubiquitinated proteins, are major components of these aggregates (Perry et al., 1987). Amyloid-Beta, produced by the cleavage of the Amyloid Precursor Protein (APP), is thought to have numerous deleterious effects on neuronal health and connectivity (Thinakaran and Koo, 2008). Mutations in the genes encoding APP or the presenilin protease enzymes that cleave APP to generate Amyloid-Beta cause early-onset AD, and support the notion that Amyloid-Beta metabolism is a central component of AD pathogenesis (Bertram et al., 2010). The generation of hyper-phosphorylated Tau is less clearly understood. It has been suggested that Amyloid-Beta is able to stimulate the kinase GSK3-B, resulting in the aberrant phosphorylation of Tau (Hernandez et al., 2010). The mechanisms governing the synthesis, processing, and degradation of these proteins remain incompletely understood. Inhibition of the proteasome causes an increase in Amyloid-Beta (Kumar et al., 2007), and numerous studies have begun to describe an extensive role for both ubiquitination and the proteasome in the production and handling of APP, Amyloid-Beta, and Tau (Figure 2).

APP is produced in the endoplasmic reticulum (ER). HRD1, an E3 ligase associated with the clearance of newly synthesized proteins through ER-associated degradation (ERAD), has been shown to interact with APP. Decreasing HRD1 expression evokes ER stress and apoptosis, accompanied by the accumulation of APP and Amyloid Beta. The disease relevance of the HRD1/APP relationship is supported by the reduced levels of HRD1 in AD brain tissue (Kaneko et al., 2010). Similarly, the levels of Fbxo2, a brain-enriched E3 ligase substrate adaptor protein also implicated in ERAD, have been reported to be decreased in AD patient tissues (Gong et al., 2010). Fbxo2 was also found to facilitate the degradation of APP, and a knockout mouse model for Fbxo2 revealed elevated levels of APP and Amyloid Beta in a brain region-specific manner (Atkin et al., 2014).

In the Golgi apparatus, APP is ubiquitinated by unknown E3 ligases stimulated by ubiquilin-1, a protein with chaperone-like properties. This ubiquitination is K63-linked and does not cause the degradation of APP. Instead, it causes the retention of APP in the early secretory pathway, impairing its maturation and delaying its subsequent processing by secretases into Amyloid Beta (El Ayadi et al., 2012). The process by which this ubiquitination of APP is normally removed to allow processing remains unclear. Ubiquilin-1 levels are significantly decreased in AD patient brain tissues, further suggesting a role in AD pathogenesis (El Ayadi et al., 2012). Single-nucleotide polymorphisms (SNPs) in the UBQLN1 gene have recently been linked to late-onset AD (Stieren et al., 2011).

After being produced and trafficked to the surface, APP is internalized via endocytosis into the endosome and trans-Golgi network and sequentially cleaved to produce Amyloid-Beta (Thinakaran and Koo, 2008). The C-terminus of HSP70 Interacting Protein (CHIP) is an E3 ligase previously associated with polyglutamine neurodegenerative disorders (Miller et al., 2005; Williams et al., 2009). In AD models, CHIP has been shown to interact with Amyloid-Beta in the Golgi, in a manner that increases upon inhibition of the proteasome. Over-expression of CHIP results in a decrease in Amyloid-Beta levels and may stabilize levels of APP (Kumar et al., 2007).

Beyond targeting APP or Amyloid-Beta directly, the USS affects numerous other components of the Amyloid pathway, including the secretase enzymes responsible for the production of Amyloid Beta. FBXW7 (SEL-10) facilitates the ubiquitination of the gamma-secretase component Presenilin 1 (PS1), although this ubiquitination unexpectedly increases Amyloid Beta production through mechanisms that remain unclear (Li et al., 2002). Fbxo2, described above, has also been implicated in the turnover of the Beta-secretase protein BACE1 (Gong et al., 2010). Similarly, overexpression of the E3 ligase Parkin, described below for its role in PD, has been shown to reduce APP expression, Amyloid-Beta burden, and inflammation while also restoring the formation of long-term hippocampal synaptic potentiation in an AD mouse model (Hong et al., 2014).

The effects of Amyloid-Beta on neurons are numerous, and can also influenced by the complex actions of the USS in more indirect methods than just the degradation of the Amyloid-Beta peptide or its precursor. For example, exposure to excess Amyloid-Beta causes diminished Brain-Derived Neurotrophic Factor (BDNF) signaling in AD patient tissue and mouse models (Peng et al., 2005); however, this diminution can be reversed by the overexpression of the de-ubiquitinating enzyme UCHL-1, which, through an unknown mechanism, restores the trafficking of BDNF receptors (Poon et al., 2013). Intriguingly, UCHL-1 levels are decreased in AD patient brains (Poon et al., 2013).

As another example, the intracellular response to Amyloid-Beta is thought to be mediated through numerous proteins including GSK3B, a kinase that is reportedly increased in AD and has Tau as one of its substrates. GSK3B has been investigated as a possible link between the two characteristic protein pathologies of AD (Hernandez et al., 2010). The kinase activity of GSK3B is enhanced by forming a complex with the tumor suppressor protein p53. Under normal conditions, this interaction is limited by the degradation of p53 by the E3 ligase Mouse double minute 2 homolog (MDM2). Under conditions of cellular stress, however, MDM2 levels are decreased, leading to the increased association of GSK3B and p53. This enhancement is thought to contribute to the hyperphosphorylation of Tau seen in AD (Proctor and Gray, 2010). The observed decrease in MDM2 under conditions of stress actually stems from its own governance by the UPS. Under normal conditions, MDM2 is modified by the small, ubiquitin-like modifier protein SUMO. Sumoylation of MDM2 prevents its auto-ubiquitination. Under conditions of cellular stress, sumoylation of MDM2 is diminished, causing it to become auto-ubiquitinated which then triggers its own degradation by the proteasome (Buschmann et al., 2000). The level of GSK3B in neurons is also mediated by yet another E3-ligase, Nedd8 ultimate buster 1 (NUB1). NUB1 directly binds to GSK3B and promotes its degradation by the proteasome, while also inhibiting the interaction between GSK3B and Tau. NUB1 activity thereby diminishes the levels of hyper-phosphorylated Tau and Tau aggregates (Richet et al., 2012).

NUB1 was originally identified for its role in regulating Nedd8 (Kamitani et al., 2001; Kito et al., 2001). Nedd8 is a small signaling protein similar to ubiquitin both in structure (approximately 60% identity and 80% homology to human ubiquitin) and in function (Kumar et al., 1993; Kamitani et al., 2001). The conjugation of Nedd 8, “neddylation,” to cullin proteins promotes the ubiquitination activity of SCF complexes (Duda et al., 2008). Nedd8 also modifies other proteins, including transmembrane proteins such as APP, and may influence their degradation (Chen et al., 2012). Dysregulated clearance of Neddylated proteins in disease states is evinced by the accumulation of Nedd8 in ubiquitin-positive tau filamentous inclusions in some cases of AD and in Lewy bodies in PD (Dil Kuazi et al., 2003).

CHIP, described above for its role in the turnover of Amyloid-Beta, has also been shown to play a role in regulating phosphorylated Tau. Through its interaction with two heat-shock-induced proteins, Hsp70 and Hsp90, CHIP is able to ubiquitinate phosphorylated Tau. Under normal conditions, this ubiquitination leads to an accumulation of ubiquitinated tau, which then becomes aggregated into high molecular-weight, detergent-insoluble aggregates. CHIP immunoreactivity decorates NFTs in several tauopathies, including AD (Petrucelli et al., 2004). However, over-expression of Hsp70 shifts the handling of ubiquitinated Tau toward a pathway of clearance through the UPS, rather than aggregation, and reduces Tau levels *in vitro* (Petrucelli et al., 2004). Intriguingly, *in vitro* the ubiquitination of Tau also is carried out in an E3-ligase independent manner by the E2 Ube2w (Scaglione et al., 2013). Ube2w, whose levels are increased under cellular stress, also ubiquitinates CHIP and regulates its activity (Scaglione et al., 2011). Additionally, a role for CHIP in the ubiquitin-mediated turnover of caspases has been described. Mice that lack CHIP show increased levels of cleaved and uncleaved caspase-3 (Dickey et al., 2006).

Although not considered causative themselves, additional proteins have been shown to exacerbate disease pathogenesis, and are similarly subject to regulation by ubiquitination. NMDA receptors are ionotropic glutamate receptors whose regulated conductance of calcium into postsynaptic sites has been linked to learning and memory. Aberrant activation and signaling of these receptors has been implicated in numerous disease processes, including AD (Hardingham and Bading, 2010). It has been suggested that the Amyloid-Beta-induced loss of synapses requires the activation of extra-synaptic NMDA receptors containing the GluN2B subunit (Shankar et al., 2007; Talantova et al., 2013), underscoring the importance of regulating NMDA receptor levels and localization in AD pathology. GluN2B is ubiquitinated in response to synaptic activity by the E3 ligase Mind Bomb-2 (Jurd et al., 2008). Fbxo2, described above for its regulation of APP, also regulates the levels of NMDA receptors *in vitro* by facilitating the activity-dependent ubiquitination and elimination of the NMDA receptor subunit GluN1 (Kato et al., 2005).

The downstream effects of NMDA receptor activation on cell health include the regulation of ubiquitin-dependent pathways. In one example, following NMDA receptor activation, the cyclin-dependent kinase 5 (Cdk5) phosphorylates Cdh1, a key regulator of the E3 ligase complex APC; in doing so, the turnover of its cyclin B1 by APC is inhibited, promoting neurotoxicity following NMDA receptor activation (Maestre et al., 2008).

### Parkinson's disease

**Figure 3:**
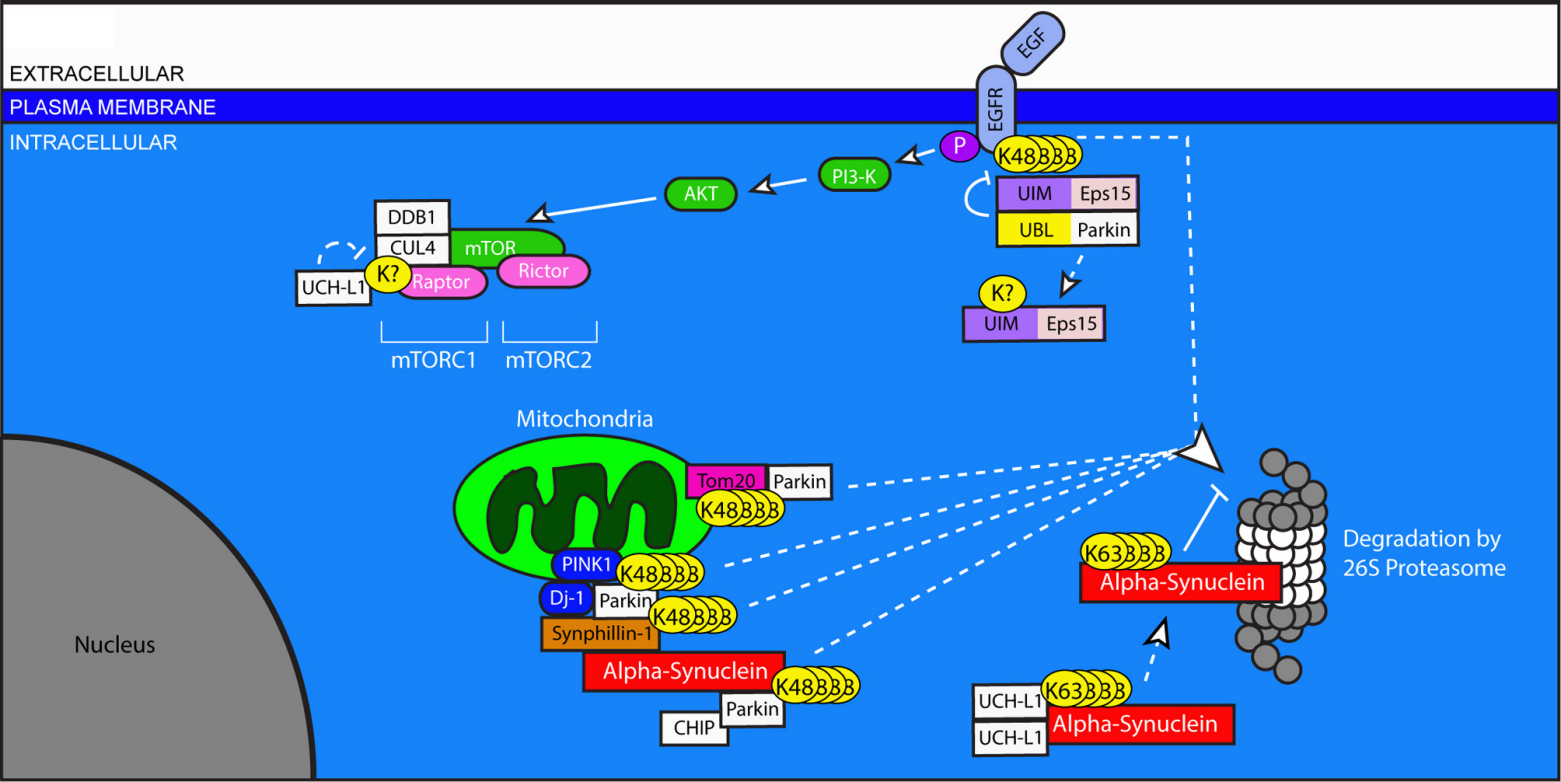
**Ubiquitination in pro-survival pathways, mitochondria stability, and protein clearance in Parkinson's disease**. Dysregulation of Parkin and UCHL1, both of which are associated with PD, has widespread effects on neuronal health. The Epidermal Growth Factor (EGF) binds to EGFR and initiates pro-survival signaling through the Akt and mTOR pathways. Ligand-binding to EGFR stimulates its ubiquitination, which allows for its UIM-dependent recognition by Eps15. Eps15 internalizes EGFR, allowing it to be trafficked to the proteasome for degradation. The EGFR-Eps15 interaction is blocked by the interaction of the UBL of the E3 ligase Parkin with the UIM of Eps15; this interaction increases Parkin's ligase activity, causing Eps15 to become ubiquitinated and dissociated from its UIM-dependent interactors. Downstream of EGFR signaling, mTOR's participation in the protein complex mTORC1 requires the ubiquitination of Raptor by DDB1 and Cul4. This ubiquitination is undone by the de-ubiquitinating enzyme UCHL1. Parkin also plays a role in the degradation of mitochondrial outer membrane proteins including Tom20, and through its involvement with PINK1 and Dj-1, facilitates the degradation of Synphilin-1 and itself. Synphilin interacts with alpha-synuclein, which can be degraded by Parkin in association with CHIP. Dimerized UCHL1 may also modify alpha-synuclein, though the result is non-degradative, K-63 linked ubiquitination which promotes its accumulation. Accumulated alpha-synuclein binds to the 20S core of the proteasome and inhibits proteasome function.

Parkinson's disease is the second most common neurodegenerative disease and the most common neurodegenerative movement disorder. It is characterized by progressive abnormalities in gait and posture, as well as difficulty initiating and completing voluntary and involuntary movements. Histopathologically, PD involves the loss of dopaminergic neurons of the substantia nigra and locus ceruleus accompanied by astrocytosis and increased numbers of microglia. Throughout the brainstem of PD patients, proteinaceous intracellular aggregates called Lewy bodies can be found, comprised primarily of alpha-synuclein. With slightly altered appearance, these synuclein-containing aggregates can be seen in other brain regions, and the question of their contribution to disease pathogenesis remains an area of intense study (Syme et al., 2002). Although the majority of PD cases are of sporadic origin, the small fraction of inherited cases has provided valuable insight into the role of the USS and UPS in PD pathology (Figure 3).

Mutations in alpha-synuclein have been described in cases of familial PD (Kruger et al., 1998; Riess et al., 1998), and the proteasome is at least partially responsible for the turnover of this protein (Shimura et al., 2001). Proteasome dysfunction is reported in the substantia nigra in PD, and proteasomal inhibition itself can cause the formation of protein inclusions and eventual degeneration of neurons in the substantia nigra in rats, although with some inconsistency (McNaught and Jenner, 2001; McNaught et al., 2002; McNaught and Olanow, 2006). The expression of mutant alpha-synuclein induces the formation of filaments which interact directly with the 20S core of the proteasome and decrease its proteolytic activity (Lindersson et al., 2004). This deficit in proteasome function is ameliorated by the concomitant expression of the E3 ligase Parkin (Petrucelli et al., 2002).

Of patients with inherited PD, nearly 40 percent of those with an early onset of symptoms have mutations in the gene encoding Parkin (Lucking et al., 2000). First described in Japan, multiple mutations in Parkin have now been described in one or both alleles (Khan et al., 2003). While mutations in alpha-synuclein cause autosomal dominant PD, mutations in Parkin cause autosomal recessive PD. These mutations result in the loss of Parkin function, one aspect of which is to regulate the ubiquitination of alpha-synuclein. Because the accumulation of ubiquitinated alpha-synuclein is evident in both familial and sporadic PD, Parkin dysfunction may also play a role in idiopathic PD and the formation of Lewy bodies (Shimura et al., 2000; Hardy, 2003). Consistent with this view, targeted expression of mutant Parkin in disease-related brain regions of animal models yields similar pathologies to those observed in PD (Lu et al., 2009).

Parkin is also involved in the intricate regulation of pro-survival signaling through the Akt pathway by Epidermal Growth Factor (EGF) (Fallon et al., 2006). EGF-Akt signaling occurs through EGFRs which are decreased in disease-related brain regions of patients with PD (Iwakura et al., 2005). Accordingly, increased activation of EGFR in an animal model of PD prevents the loss of dopaminergic neurons (Iwakura et al., 2005). EGF signaling is regulated by ubiquitin in several ways. Ligand binding of EGFR stimulates Akt signaling, but triggers the endocytosis and degradation of EGFR, thereby limiting the extent of EGF signaling. This process is mediated by the activity-dependent ubiquitination of EGFR by an unknown E3 ligase, which makes it eligible for recognition by the ubiquitin-interacting motif (UIM) of the EGFR Protein tyrosine kinase Substrate #15, Eps15. Interaction with Eps15 is necessary for the internalization of EGFR, as Eps15 links ubiquitinated EGFRs to the machinery which traffics them to the proteasome for degradation (van Bergen En Henegouwen, 2009). Parkin, which contains an amino-terminal ubiquitin-like (UBL) domain, blocks the Eps15-EGFR interaction by binding to the UIM of Eps15 through this UBL domain, and then facilitating the ubiquitination of Eps15. In this manner, EGFR internalization is prevented, and EGF signaling and activation of pro-survival pathways are enhanced. But interaction between Eps-15 and Parkin suggests yet a further level of ubiquitin-dependent regulation. Eps-15 promotes its own ubiquitination by stimulating Parkin's E3 ligase activity upon interaction. This ubiquitination, which is believed to be K63-linked, prevents the interaction of ubiquitinated Eps15 with other ubiquitinated proteins such as EGFR and Parkin itself. Notably, Parkin deficiency causes a reduction in EGF signaling (Fallon et al., 2006). Here, again, dysfunctional ubiquitin signaling, independent of proteasomal cleavage, is able to regulate the health of neurons in a disease context.

Parkin has been shown to function as part of a macromolecular E3 ligase complex which includes the pten-induced kinase (PINK1) and the peptidase Dj-1 (Xiong et al., 2009). Mutations in both PINK1 and Dj-1 cause hereditary PD (Abou-Sleiman et al., 2003; Valente et al., 2004). Together, these proteins facilitate the degradation of Parkin substrates. Synphillin-1, which interacts with alpha-synuclein and is also present in Lewy bodies, is one such substrate, as is Parkin itself (Xiong et al., 2009). Additionally, the interaction between PINK1 and Parkin facilitates the degradation and turnover of mitochondria (Greene et al., 2003; Vincow et al., 2013). In order to do so, Parkin targets proteins in the outer mitochondrial membrane for degradation, including Tom20 (Yoshii et al., 2011). These findings suggest that Parkin dysfunction may contribute to global cellular health problems, even beyond the accumulation of alpha-synuclein and synphillin-1.

Rare mutations associated with familial, early-onset PD are also found in the de-ubiquitinating enzyme UCH-L1, described above for its role in AD (Das et al., 2006). These disease-associated mutations reduce the DUB activity of UCHL1. UCH-L1's role is significant for the health and function of neurons through its regulation of substrates involved in governing mRNA transcription, protein translation, synaptic plasticity, and pro-survival signaling. These processes are potently influenced by the activity of a serine/threonine protein kinase originally identified for its susceptibility to inhibition by Rapamycin, the Mammalian Target of Rapamycin, mTOR (Sabatini et al., 1994; Hay and Sonenberg, 2004; Ghosh et al., 2008; Hoeffer and Klann, 2010). Intriguingly, mTOR function requires both the correct ubiquitination of interacting proteins and proteasome activity (Ghosh et al., 2008; Quy et al., 2013). mTOR asserts its effects through the formation of large protein complexes. mTOR can function in combination with Raptor (mTOR Complex 1 or mTORC1) or Rictor (mTORC2). mTORC1 regulates transcription and is important for local protein synthesis, a necessary component of several types of synaptic plasticity. mTORC2, on the other hand, influences Akt signaling, cytoskeletal dynamics, and actin polymerization (Costa-Mattioli and Monteggia, 2013). The balance between mTORC1 and mTORC2 formation depends on the interaction of Raptor with a multi-subunit E3 ligase composed of DNA Damage-Binding Protein (DDB1), Cullin 4A (CUL4), and the RING-type E3 ligase RING box 1 (RBX1) (Ghosh et al., 2008). Although the exact mechanism is still unclear, the DDB1-CUL4 complex appears to ubiquitinate Raptor in a manner that facilitates its incorporation into the mTORC1 complex including DDB1, CUL4, Raptor, and mTOR (Ghosh et al., 2008). UCHL1 deubiquitinates Raptor, thereby destabilizing mTORC1 complex formation and shifting the balance toward mTORC2-dependent signaling (Hussain et al., 2013). This shift may have a significant impact on the ability of neurons to modify their synaptic content to promote learning and memory, and may also represent a neuroprotective response mechanism.

The loss of UCHL1's DUB activity results in an accumulation of alpha-synuclein at presynaptic terminals rather than increased clearance (Cartier et al., 2012). Surprisingly, UCH-L1 had been proposed to act as an E3 ligase when it self-dimerizes; this new activity is independent of the state of UCHL1's DUB activity (Liu et al., 2002). Acting as an E3 ligase, UCHL1 causes the K63-linked ubiquitination of alpha-synuclein, which, in turn, worsens PD pathology (Liu et al., 2002). Accordingly, a mutant form of UCH-L1 with decreased E3 ligase activity upon dimerization, but normal DUB activity, decreases PD pathogenesis (Liu et al., 2002). The consequences of UCHL1's ubiquitination of alpha-synuclein remain unclear.

There have been several ubiquitin-linked proteins whose involvement in neurotoxicity overlaps between AD and PD. Of those described above, CHIP, MDM2, and HRD1 support the importance of the UPS in pathological mechanisms in multiple disease states. CHIP also interacts with Parkin and stimulates its ligase activity (Imai et al., 2002). Even without additional insult, the down-regulation of MDM2 is toxic to dopaminergic neurons (Nair et al., 2006). And HRD1 levels are increased in dopaminergic neurons under conditions of neurotoxicity, suggesting a role in the response to cellular stress (Mei and Niu, 2010).

### Amyotrophic Lateral Sclerosis

**Figure 4:**
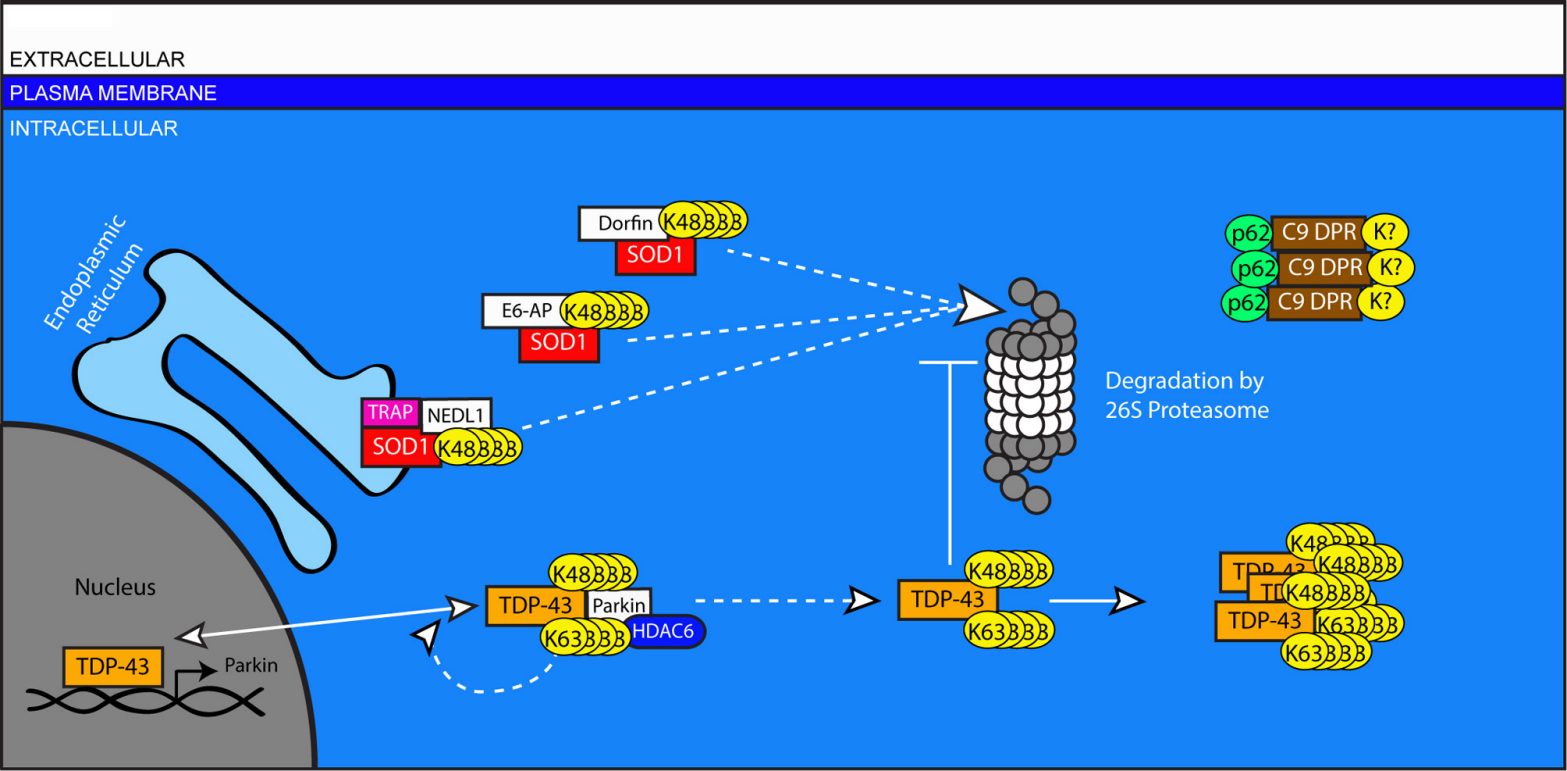
**A role for ubiquitination in the pathogenesis of Amyotrophic Lateral Sclerosis**. Impaired turnover of SOD1 and TPD-43 are implicated in the etiology of ALS. Mutated or excess SOD1 in the ER is targeted for degradation by the E3 ligase NEDL1 via the translocon protein TRAP. SOD1 in the cytosol is targeted by the canonical HECT-type E3 ligase, E6-AP, and the RING-type E3 Dorfin. TDP-43 can be ubiquitinated by Parkin in association with HDAC6. This modification can include both K48 and K63-linked chains and does not lead to the degradation of TDP-43, instead increasing the proportion of TDP-43 in the cytosol. In the nucleus, TDP-43 regulates the transcription of genes including Parkin. In the cytosol, accumulated TDP-43 can inhibit the proteasome and form protein aggregates. In cases associated with mutations in the C9ORF72 gene, aggregates of DPR proteins are present with ubiquitin- and p62-positive immunoreactivity, but do no colocalize with TPD-43.

Amyotrophic Lateral Sclerosis (ALS) is the most common fatal neurodegenerative disease affecting motor neurons. The loss of motor neurons in the brain and spinal cord of ALS patients typically results in progressive paralysis and death within a few years of onset. Non-motor pathologies, particularly cognitive deficits, can also accompany motor deficits. Histopathologically, ubiquitin-rich cytoplasmic inclusions appear in the remaining motor neurons of ALS patients, accompanied by marked gliosis. Ubiquitin-positive inclusions can also be observed in cortical brain regions, consistent with the observed cognitive decline in many ALS patients (Lowe, 1994; Walling, 1999). Sporadic ALS (SALS) represents greater than 90 percent of all ALS cases, with familial ALS (FALS) accounting for the remaining <10 percent. The etiology of ALS remains unclear, and what is known to date has been determined largely through the study of genes originally identified as mutated in FALS. Importantly, the disease genes that cause FALS are also found mutated in some SALS cases, and the histopathologies of familial and sporadic cases are essentially indistinguishable (Andersen and Al-Chalabi, 2011), suggesting that studies of FALS will offer useful insight into all forms of ALS. Among the disease genes associated with FALS, mutations in the gene encoding Superoxide Dismutase 1 (SOD1) were described first (Rosen et al., 1993), and mutations in the Transactivation Response DNA-binding protein 43 (TDP-43) are among the most common (Gitcho et al., 2008). Recently, genome-wide association studies revealed a large hexanucleotide expansion—upwards of 1600 repeats—within the non-coding region of the C9ORF72 gene to be causative in a Finnish cohort (Laaksovirta et al., 2010). Numerous other studies have now observed this expansion in 20–50 percent of FALS cases, making it the most common cause (Boeve et al., 2012; Chio et al., 2012; Simon-Sanchez et al., 2012). The protein products of these genes accumulate in ubiquitin-positive inclusions in disease-related neuronal populations, and the mechanisms of their turnover and clearance provide insight into the importance of the UPS in ALS (Figure 4).

SOD1 plays an important role in the elimination of free superoxide radicals. Its expression is regulated by several agents of the UPS. The canonical HECT-type E3 ligase E6-AP directly binds to and ubiquitinates SOD1 (Mishra et al., 2013). Levels of E6-AP are decreased prior to the onset of neurodegeneration in a mouse model of ALS expressing mutant SOD1, and over-expression of E6-AP reduces the aggregation of mutant SOD1 *in vitro* (Mishra et al., 2013). NEDL1, a homolog of E6-AP, is reported to selectively bind to mutant SOD1 but not wild-type SOD1, facilitating its ubiquitination and clearance (Miyazaki et al., 2004). NEDL1 is thought to function in concert with the endoplasmic reticulum translocon-associated protein TRAP-δ, suggesting a role for NEDL1in the ERAD of mutant SOD1 (Kunst et al., 1997). The interaction between NEDL1 and mutant SOD1 increases with disease severity, and NEDL1 immunoreactivity is observed in SOD1-positive inclusions in human spinal cord tissue from FALS patients (Miyazaki et al., 2004). Also reportedly present in SOD1-positive inclusions is the RING-finger type E3 ligase Dorfin, which shares NEDL1's selectivity in ubiquitinating mutant, but not wild-type, SOD1 (Niwa et al., 2002). Over-Expression of Dorfin in a mouse model of FALS reduces expression and aggregation of SOD1 while also diminishing motor neuron degeneration (Sone et al., 2010).

The protein TDP-43, extensively aggregated in many in ALS cases, normally has a predominantly nuclear localization. It has two RNA binding domains and has been implicated in RNA splicing and trafficking (Buratti and Baralle, 2001, 2010). Ubiquitinated TDP-43 is a major component of cytoplasmic inclusions in ALS (Neumann et al., 2006). Mutations associated with ALS result in the redistribution of TDP-43 from the nucleus to the cytoplasm (Igaz et al., 2011), although the precise mechanisms by which TDP-43 contributes to ALS pathogenesis remain the subject of intensive study (Janssens and Van Broeckhoven, 2013). TDP-43 expression inhibits proteasome function and increases levels of Parkin mRNA and protein (Hebron et al., 2013) by binding to Parkin mRNA (Polymenidou et al., 2011). Parkin, in turn, is able to mediate both K48 and K63-linked ubiquitination of TDP-43, though this modification does not cause a reduction in the levels of TDP-43 (Hebron et al., 2013). TDP-43 is unlike other substrates of Parkin-dependent K63-linked ubiquitination, which are targeted for degradation through the autophagic pathway (Olzmann and Chin, 2008). The interaction between TDP-43 and Parkin requires the histone deacetylase protein HDAC6 and promotes the translocation of TDP-43 from the nucleus to the cytoplasm (Hebron et al., 2013).

In C9ORF72-related ALS cases, TDP-43 is similarly present in ubiquitin-positive, cytoplasmic aggregates (Mackenzie et al., 2014). However, a second ubiquitin-positive pathology is also observed: ubiquitin- and p62-positive inclusions that are TDP-43 negative, but stain positive for dipeptide products of abnormal transcripts from the hexanucleotide repeats in C9ORF72 (Mackenzie et al., 2014). These dipeptide-repeat (DPR) products are made by unconventional, repeat-associated, non-ATG initiated (RAN) translation of transcripts containing the hexanucleotide repeat expansion (Ash et al., 2013). The pathogenic contribution of these dipeptide products is as yet unclear, but their extensive colocalization with ubiquitin and p62 may suggest a failed attempt by the cells to clear them and the toxic response they may evoke (van Blitterswijk et al., 2012).

### Huntington's disease

Huntington's disease is the most common of the polyglutamine (polyQ) disorders. While polyglutamine repeats are common motifs that may facilitate protein-protein interactions, expansion of these repeats is associated with at least nine different neurodegenerative disorders (Ross, 1995; Orr, 2012). The threshold length at which this expansion begins to elicit adverse effects varies among these diseases, determined in part by the specific protein context neighboring the repeat in the expanded protein (Robertson et al., 2011). Expansion of the CAG repeat in exon 1 of the gene coding for the Huntingtin protein increases the length of the polyglutamine domain (Myers et al., 1993; Snell et al., 1993). The repeat length threshold for disease in HD is 38 or more repeats (Chong et al., 1997), with longer repeats causing earlier onset and more severe disease. Symptoms of HD include progressive cognitive impairment, mood disorder and other psychiatric symptoms, and highly disordered movement and motor control, including abnormal movements such as chorea. These progressive symptoms lead to mortality within 20 years of onset (Walker, 2007). Histopathologically, HD is characterized by the degeneration of striatal and cortical neurons and the presence of intraneuronal, intranuclear aggregates, and dystrophic neurites. Both of these latter features are marked by the presence of ubiquitinated Huntingtin. These aggregates can be found in cortical and striatal neurons, with some variance in cortical localization related to the age of onset (DiFiglia et al., 1997).

**Figure 5:**
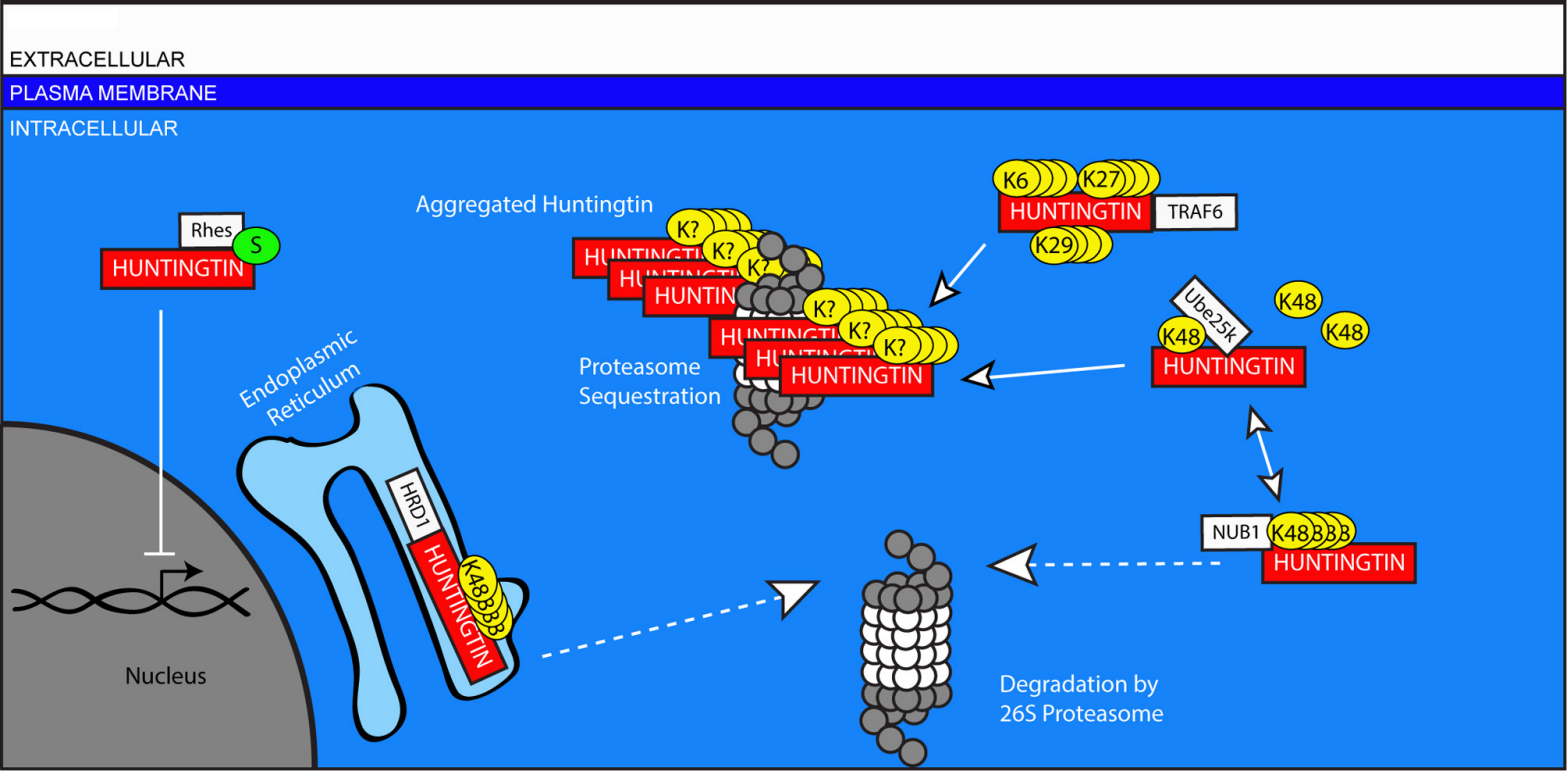
**Ubiquitin-mediated handling of the pathologic Huntingtin protein in Huntington's disease**. The Huntingtin protein is improperly cleared from neurons in HD. Mutant Huntingtin can be targeted for degradation in the ER by HRD1, and in the cytosol by NUB1. However, this ubiquitination can be edited by UCHL1, inhibiting the degradation of Huntingtin and promoting its aggregation in the cytosol. Traf6 facilitates the non-canonical ubiquitination of Huntingtin through the formation of K6, K27, and K29-linked polyubiquitin chains, and these modifications selectively promote the aggregation of mutant Huntingtin. Aggregates of mutant Huntingtin include components of the UPS including proteasomes. Rhes promotes the sumoylation of Huntingtin, which reduces its aggregation, but causes sumoylated, mutant Huntingtin to interfere with other cellular processes including gene transcription in the nucleus.

The normal function of Huntingtin remains unknown. Structural analyses and numerous other studies have led scientists to propose several functions including intracellular protein trafficking, modulation of gene transcription, and regulation of scaffolding proteins and NMDA receptors at the synapse (Cattaneo et al., 2005; Parsons et al., 2013). NMDA receptors are improperly localized to extra-synaptic sites in HD, and their signaling at those sites contributes to the onset of symptoms in HD animal models (Milnerwood et al., 2010; Gladding et al., 2012). This mislocalization decreases synaptic NMDA receptor activation, which has been shown to promote Huntingtin aggregation and neuronal survival (Okamoto et al., 2009). Whether HD arises exclusively from a toxic gain of function for mutant Huntingtin or from an additional partial loss of normal Huntingtin function remains unclear. In either case, ubiquitin plays a significant role in the handling of normal and mutant Huntingtin protein (Figure 5).

The presence of ubiquitinated Huntingtin suggests a failure of the UPS. Huntingtin is ubiquitinated (Kalchman et al., 1996), but it has been proposed that the proteasome may be unable to process expanded polyglutamine stretches, resulting instead in the accumulation of peptide fragments containing polyglutamine (Venkatraman et al., 2004). The fate of these peptide fragments is unclear, but they may remain associated with the proteasome and inhibit its function (Holmberg et al., 2004; Raspe et al., 2009). Intriguingly, components of the UPS, including proteasomes, have been identified within Huntingtin aggregates (Suhr et al., 2001). This may represent deleterious proteasomal sequestration in aggregates, even though aggregation of mutant Huntingtin may itself be neuroprotective (Holmberg et al., 2004; Okamoto et al., 2009). The idea that Huntingtin-induced pathology includes a deficiency in UPS-mediated clearance is supported by the beneficial effects observed following efforts to increase proteasomal activity in models of HD (Seo et al., 2007). The upregulation of UPS agents through the action of histone deacetylase inhibitors may also improve the aggregation phenotype of HD model mice (Jia et al., 2012).

The precise mechanisms by which Huntingtin is ubiquitinated are not well understood. It is unclear whether, like TDP-43, ubiquitination of Huntingtin includes both K48 and K63 linkages. Most studies investigating this question have used models over-expressing a fragment of Huntingtin with a polyQ expansion. While such over-expression models induce aggregation and toxicity, their physiological relevance to the human disease state remains an area of some debate. That said, in these model systems several components of the UPS contribute to the clearance of mutant Huntingtin. HRD1, described above for its role in several diseases, is implicated in the clearance of mutant Huntingtin. The activity of HRD1 increases with Huntingtin polyQ expansion length, suggesting that HRD1 regulation may not typically handle normal, unexpanded Huntingtin (Yang et al., 2007). The Tumor Necrosis Receptor Associated Factor 6 (TRAF6) is an E3-ligase which binds to both unexpanded and mutant Huntingtin and facilitates non-canonical ubiquitination through K6, K27, and K29-linked chains (Zucchelli et al., 2011). The physiological role of this modification is unclear, but it promotes the aggregation of mutant Huntingtin without changing the localization of the wild-type protein. Additionally, NUB1, described above for its role in AD, works in conjunction with Cullin 3 to facilitate the ubiquitination and clearance of mutant Huntingtin (Lu et al., 2013).

Sumoylation also can regulate HD pathogenesis. Rhes, a striatal protein which acts as a SUMO E3 ligase, binds to mutant Huntingtin selectively and facilitates its sumoylation (Subramaniam et al., 2009). The sumoylation of mutant Huntingtin decreases its aggregation; however, disaggregated, sumoylated, mutant Huntingtin inhibits transcription, increases cytotoxicity through Caspase-3, and may impair the induction of autophagy (Choo et al., 2004; Steffan et al., 2004; Mealer et al., 2013).

Huntingtin is also reported to interact with the E2 enzyme hE2-25k (Ube2K) (Kalchman et al., 1996). The presence of Ube2k immunoreactivity in HD patient brains has been observed, and the Huntingtin-Ube2k interaction promotes aggregation and cytotoxicity in a manner that requires E2 catalytic function (de Pril et al., 2007). Ube2k has been shown to interact with numerous RING-finger E3 ligases (Lee et al., 2001). It is possible that Ube25k cleaves the polyubiquitin chains attached to Huntingtin to an extent that degradation is no longer signaled.

## Conclusions: too big not to fail

Befitting its extensive contributions to a wide range of cellular processes, the systems of protein ubiquitination and ubiquitin-dependent clearance are implicated at many levels of the most common neurodegenerative disorders. Is this implied involvement in disease incidental, or does dysregulated ubiquitination and clearance represent an inevitable and common pathway for the worsening of all neurodegenerative diseases? Because alterations in ubiquitin-dependent gene transcription, translation, control of protein quality and maturation, trafficking, mitochondrial turnover, and the handling of protein-protein interactions are found in combination in neurodegenerative disease, it seems likely that dysregulated ubiquitination will not remain limited to a single ubiquitin-dependent process in a given disease. Instead, the UPS and USS will be widely and progressively involved. To what degree this involvement might begin the pathogenic cascade is currently unclear. But based on the studies reviewed here, it seems more likely that through a complex web of dysfunction in the UPS and USS, involvement of these systems causes the pathogenicity of aberrant proteins to diversify and flourish, affecting additional systems and promoting the loss of synapses and cell death. As questions remain about the mechanism by which the deposition of abnormal protein progresses through regions of a diseased brain, mounting evidence strongly supports the view that this spread occurs within the context of weakening protein quality control and ubiquitin-mediated pathways.

The UPS is not alone in its handling of unwanted or toxic proteins. The autophagic system is the other major pathway by which protein clearance is achieved. Autophagy especially regulates the turnover of organelles and aggregated protein species, and accordingly its role in neurodegenerative disease has been extensively pursued. Upon failure of the proteasome, autophagic mechanisms can be induced as a compensatory response to UPS inhibition in numerous neurodegenerative diseases (Garcia-Arencibia et al., 2010; Metcalf et al., 2012; Nixon, 2013). Further activation of autophagic mechanisms through pharmacologic or genetic manipulation has proven useful as a therapeutic intervention in model systems (Hochfeld et al., 2013). It seems evident, however, from the continued accumulation of ubiquitin-positive proteins in neurodegeneration, that autophagic induction is not sufficient to overcome this failure. Moreover, it is unclear what effect sustained, heightened autophagy might have on already weakened neurons.

The overlapping involvement of certain E3 ligases like HRD1, NUB1, and CHIP not only speaks to their significance as key regulators of proteostasis but also nominates them as important targets for research. While knocking out CHIP has been shown to exacerbate polyglutamine pathology (Williams et al., 2009), CHIP over-expression can reduce proteotoxicity and the effects of cellular stress in numerous models (Miller et al., 2005; Adachi et al., 2007; Lee et al., 2013). But CHIP induction without concurrent upregulation of Hsp70 might worsen tau pathology, suggesting that combinatorial approaches to therapy will be required.

In considering the specific goals and appropriate timing for interventions intended to prevent or delay disease onset, it is important to acknowledge that the loss of synapses can precede neurodegeneration and is thought to underlie many of the earliest cognitive impairments in numerous diseases. As such, it may represent a key step in the disease cascade and a critically important target for therapies. Alternatively, synaptic connections may simply be too costly for unhealthy neurons to properly maintain, and the loss of synaptic connections is, in effect, a response intended to limit inappropriate and potentially dangerous synaptic signaling in disease states. In the latter case, this dauer-like state is ultimately ineffective in staving off neurodegeneration, but may slow the process. With respect to human disease, perhaps restoring synapses could improve quality of life—regardless of whether such a change ultimately lengthens or shortens the life-span of affected neurons. For this reason, elucidation of the processes by which ubiquitin governs synapse formation, maintenance, and removal under normal conditions may prove invaluable.

### Conflict of interest statement

The authors declare that the research was conducted in the absence of any commercial or financial relationships that could be construed as a potential conflict of interest.
